# Supplementary material for: Controls on surface water carbonate chemistry along North American ocean margins
Source: Nat Commun. 2020 Jun 1;11:2691. doi: 10.1038/s41467-020-16530-z (PMC7264343; doi:10.1038/s41467-020-16530-z)
Supplement: Supplementary file 1 — Final SI KRF [file 41467_2020_16530_MOESM1_ESM.pdf]

Supplementary for “Controls on Surface Water Carbonate Chemistry along North American Ocean Margins” by Cai et al.

Supplementary Figures

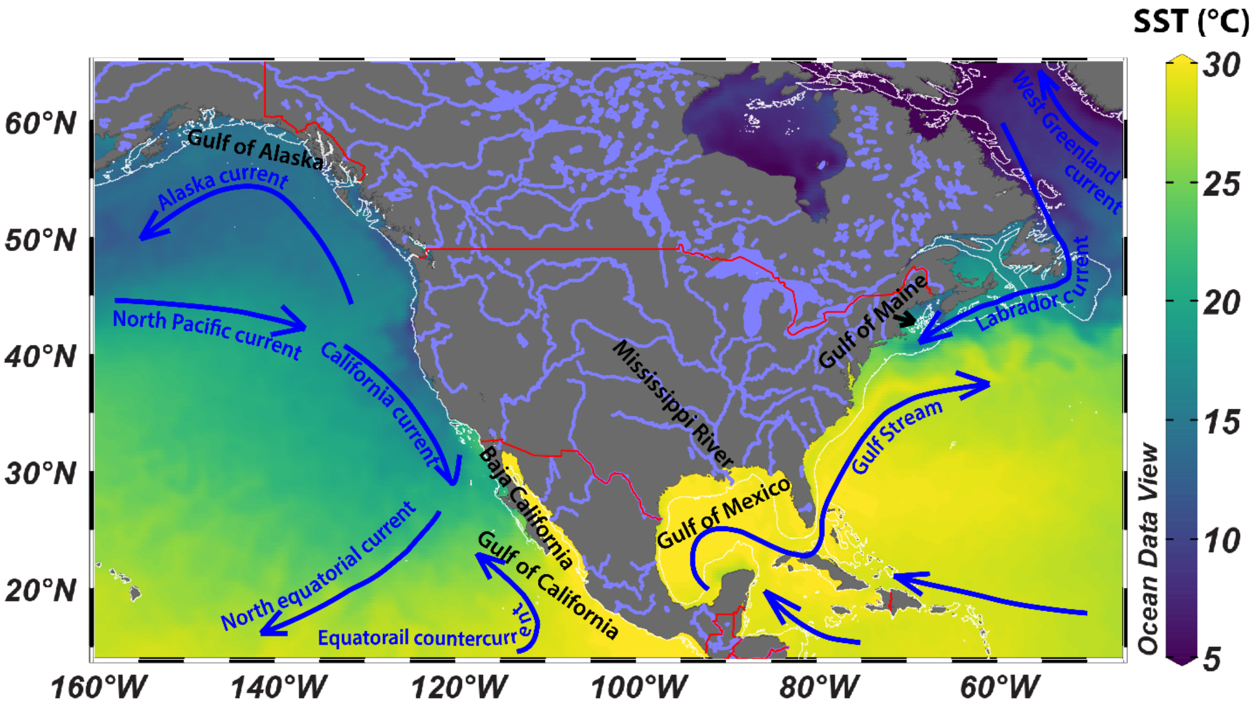

Supplementary Figure 1 | Map of the North American margins with ocean surface temperature and currents. Ocean color is the sea surface temperature (SST) for August 2016 from NOAA OI SST V2 High-Resolution Dataset (https://www.esrl.noaa.gov/psd/data/gridded/data.noaa.oisst.v2.highres.html). White dotted lines are the 200 m isobaths. Coastal ocean currents marked here and mentioned in the main text are: Gulf Stream, Labrador Current, and California Current. Locations marked here and mentioned in the main text are: the Gulf of Mexico, the Gulf of Maine, the Gulf of California and the Gulf of Alaska.

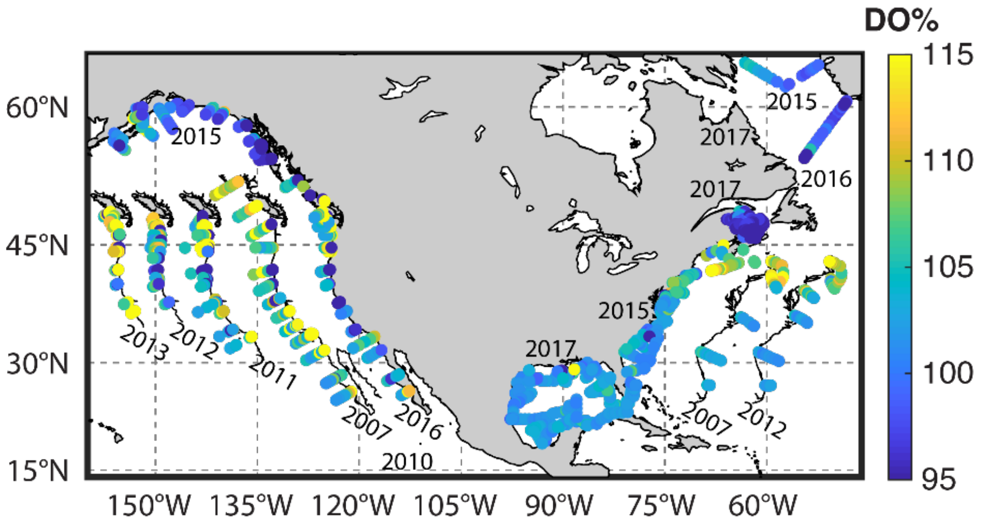

Supplementary Figure 2 | Distribution of percent oxygen saturation (DO%) in surface seawater along the North American margins. This is similar to the other distributions presented the main text Fig. 2 and Fig. 3.

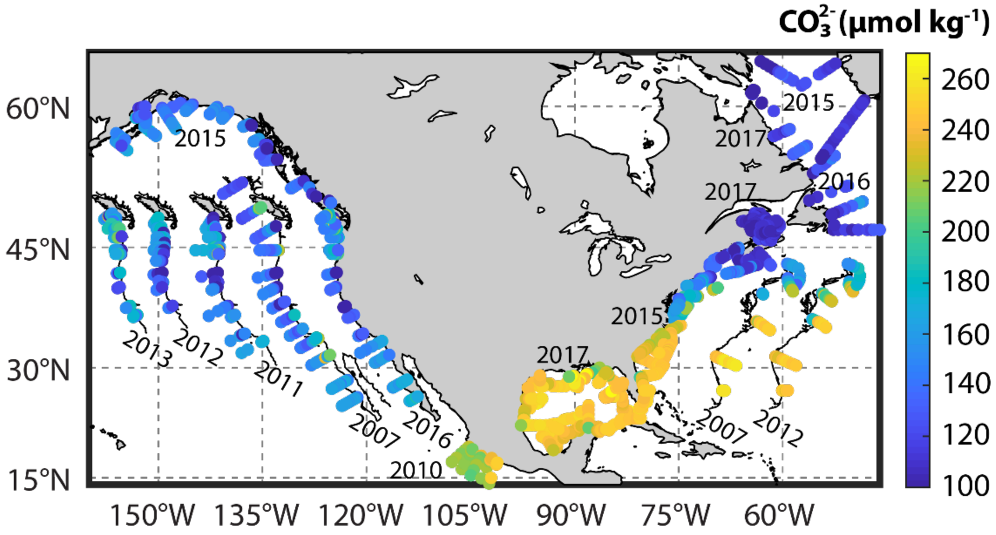

Supplementary Figure 3 | Spatial distributions [CO_3^{2-}] in the North American margins. This is similar to the other distributions presented the main text Fig. 2 and Fig. 3. In particular the distribution of Ω_{arag} is closely related to [CO_3^{2-}]. The spatial distribution of [CO_3^{2-}] is also similar to and inversely related to DIC/TA in Fig. 2f in the main text.

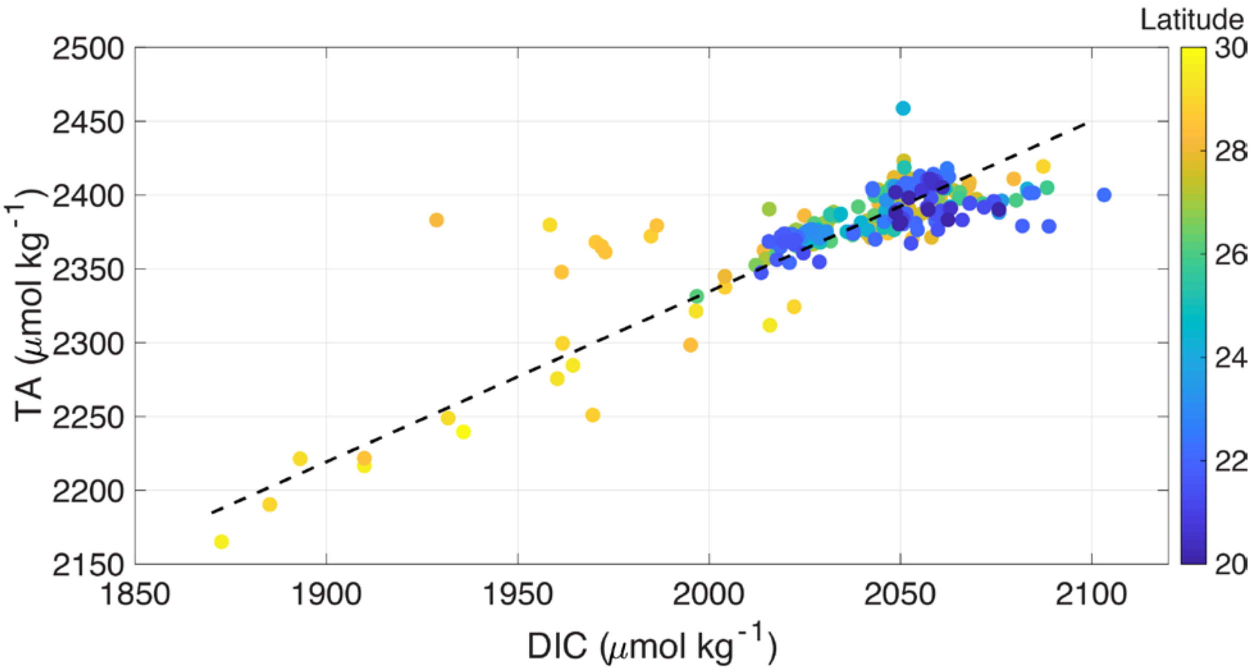

Supplementary Figure 4. A plot property-property plot of TA and DIC in the Gulf of Mexico (GOM) color-coded by latitude. In the southern GOM (lower latitudes), some lower TA relative to DIC indicates $CaCO_3$ precipitation likely occurring in waters above $CaCO_3$-rich banks at the Florida Keys and Yucatan peninsula.

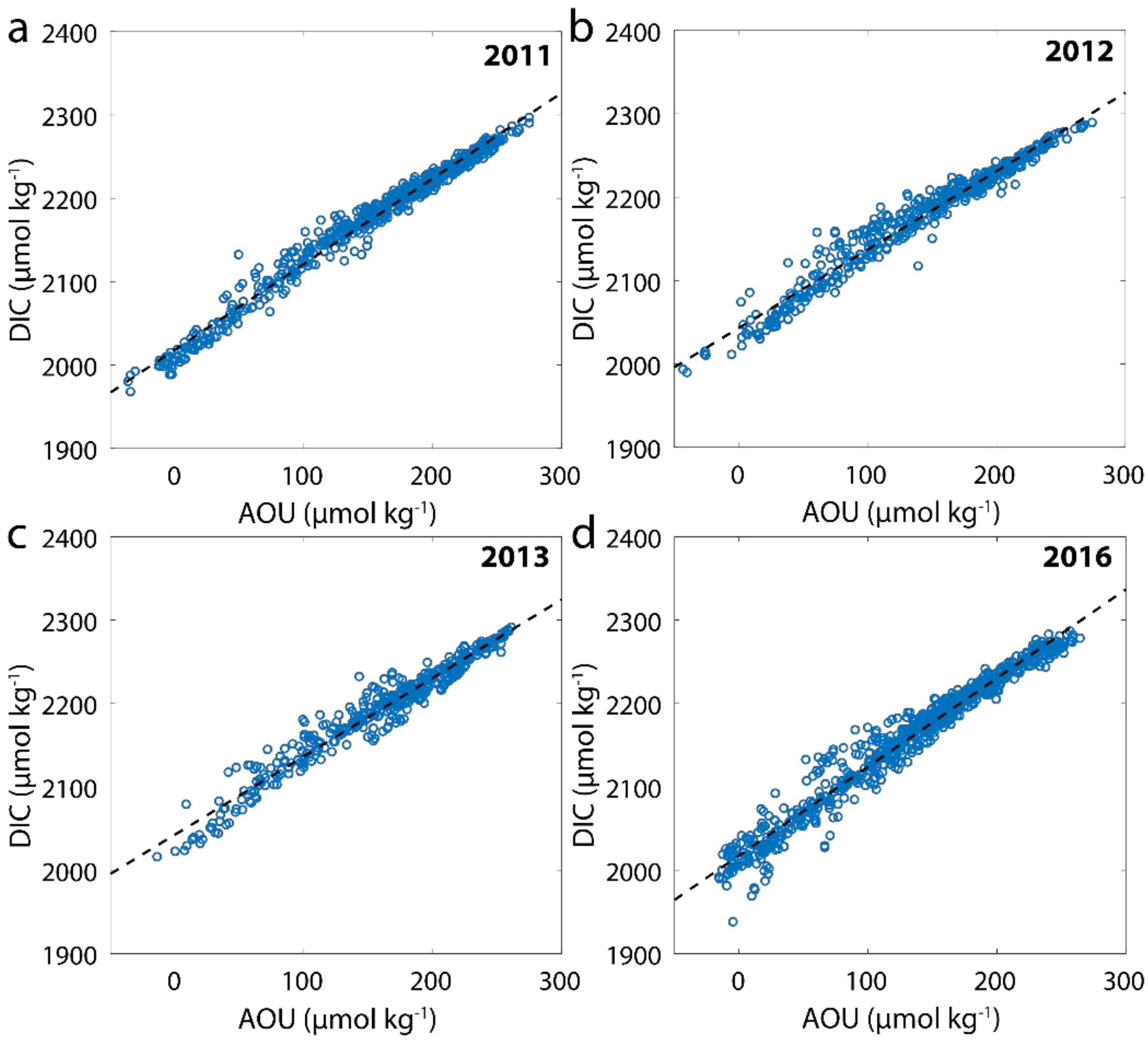

Supplementary Figure 5 | DIC and Apparent O_2 Utilization in the California Current System. The Apparent O_2 Utilization (AOU) is defined as the difference between the expected O_2 value when the water mass was previously at sea surface and in equilibrium with the atmosphere and the measured O_2 saturation concentration. AOU reflects the biological use of O_2 in the deep water. These are not the same surface water data presented in Fig. 2 in the main paper, but rather from the depths of 25-310 m. The near linear correlation reflects that the source of DIC is associated with respiration and upwelling. Previous research has shown that an anthropogenic CO_2 component was also added to the observed signal when the water mass was previously at sea surface and in equilibrium with the atmosphere.

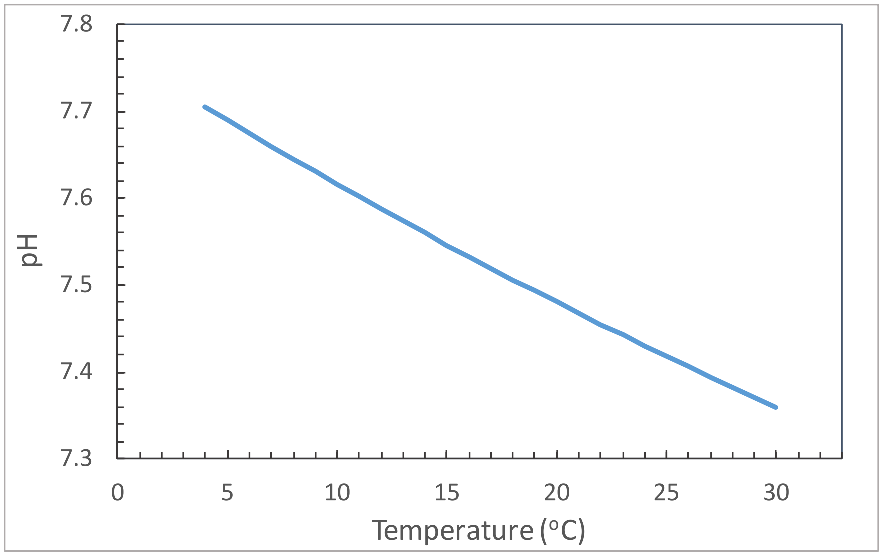

Supplementary Figure 6 | Most sensitive pH point to temperature relationship in seawater systems. This is calculated as pH = -0.5log(K_1K_2) at salinity 35. Average value is 7.53±0.18.

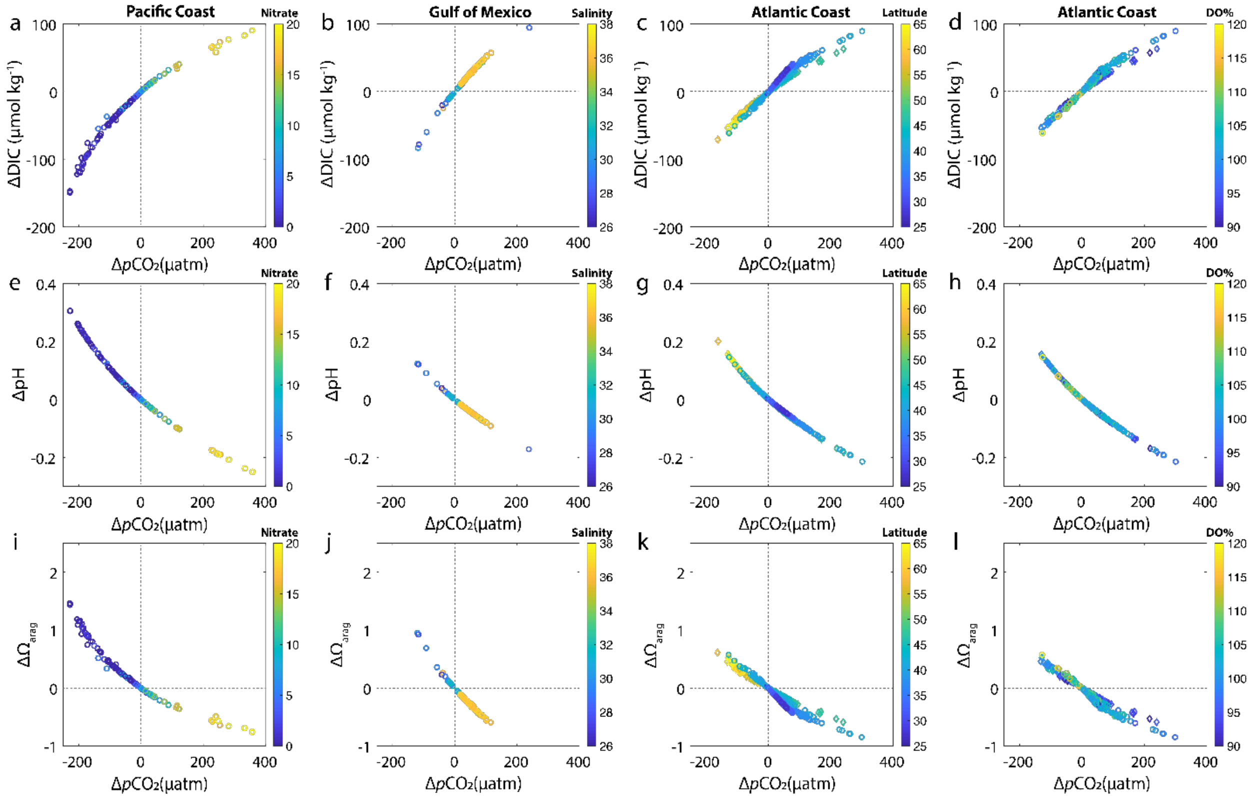

Supplementary Figure 7 | Deviations of DIC, Ω_{arag} and pH from the atmospheric equilibrium and their correlations with the deviations of water pCO$_2$ from the atmospheric pCO$_2$ (disequilibrium) in the three coasts. Nitrate concentration is used in the CCS to indicate upwelling (high) and biological production (low). Salinity is used in the Gulf of Mexico to differentiate the productive river plume (low) and the offshore waters (high). Along the Atlantic coast, both latitude and DO% are used to indicate general locations and biological production status as the controls are more complicated. Note that (c) and (d) show negative ΔDIC and ΔpCO$_2$ accompanied by high DO% in the high latitude regions (Eastern Canadian Seas).

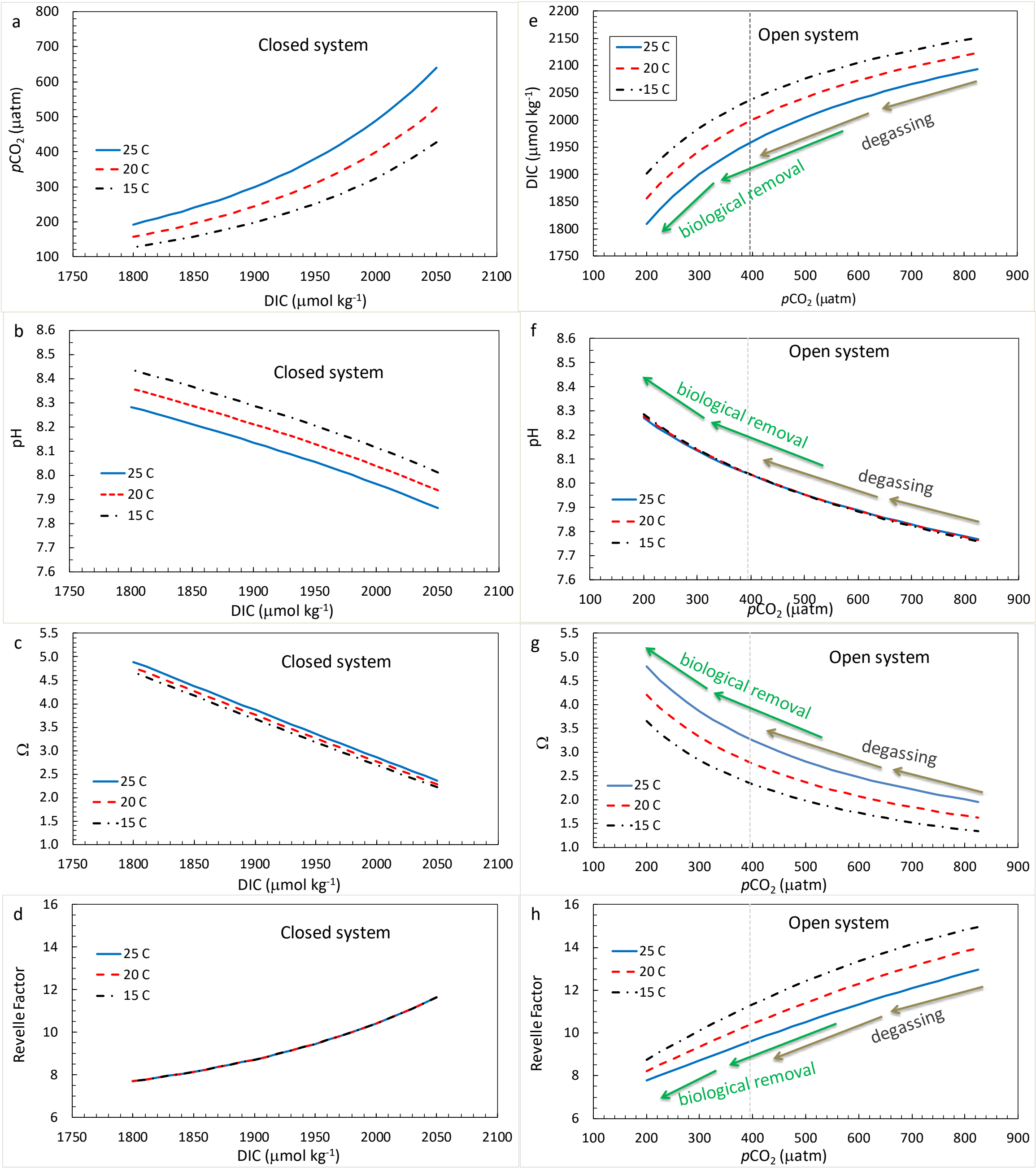

Supplementary Figure 8 | Property variations as a function of water DIC concentration and water *p*CO_2 under different temperatures. Left column: (a) *p*CO_2 vs. DIC, (b) pH vs. DIC, (c) Ω_{arag} vs. DIC, (d) Revelle Factor vs. DIC. Right column: (e) DIC vs. *p*CO_2, (f) pH vs. *p*CO_2, (g) Ω_{arag} vs. *p*CO_2, (h) Revelle Factor vs. *p*CO_2. On the left column, the calculation are done under closed system conditions similar to that in Figure 1 (TA = 2300 µmol kg^{-1}, S = 35): On the right column, the calculation is done under an open system condition similar to that in Figure 1 except *p*CO_2 values are variable (TA= 2300 µmol kg^{-1}, S = 35). A comparison of the two columns shows that as long as there is sufficient gas exchange (right) pH is not sensitive but Ω_{arag} is sensitive to temperature change. The “closed system” simulation however shows the opposite behavior.

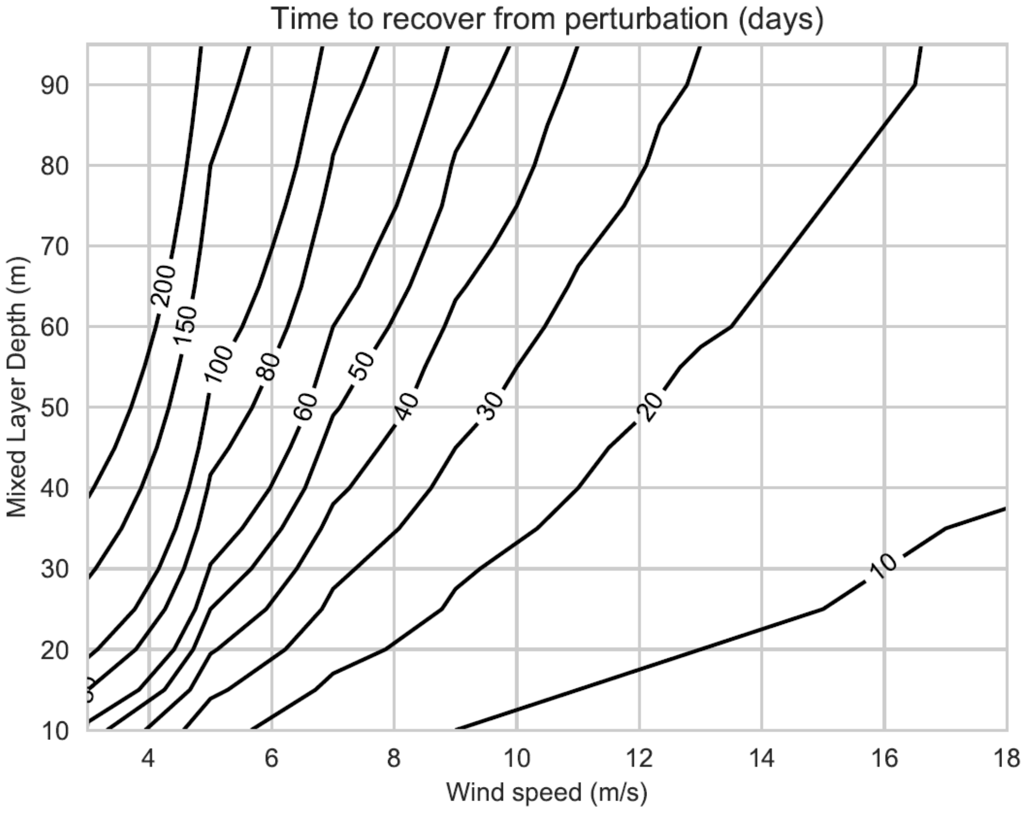

Supplementary Figure 9 | Recovery time or *e*-folding time after an instantaneous increase of 17 μmol kg^{-1} in DIC at constant TA under different wind speeds and mixed layer depths. Note that the *e*-folding time is the time interval in which an exponentially growing quantity increases or decreases by a factor of *e* (or roughly 2.7); it is the base-*e* analog of doubling time. At average wind of 7 m s^{-1} and a mixed layer depth of 30 m, the *e*-folding time is about 34 days, a time that ΔpCO_2 will decrease about 63%. Note that two to three *e*-folding times are needed for 86% to 95% of a disturbed signal to be considered as completely recovered.

Supplementary Tables

Supplementary Table 1. Cruise information.

Data Set Name	Cruise dates	Location	North Boundary	South Boundary	East Boundary	West Boundary
WCOA 2007	11 May - 14 June 2007	U.S. West Coast	52.23	24.92	-112.82	-132.82
WCOA 2011	12 - 30 August, 2011	U.S. West Coast	48.38	31.95	-117.75	-127.55
WCOA 2012	4 - 17 September, 2012	U.S. West Coast	48.38	37.67	-122.89	-126.09
WCOA 2013	3 - 29 August, 2013	U.S. West Coast	48.84	36.52	-121.85	-126.61
WCOA 2016	5 May - 7 June, 2016	U.S. West Coast	52.40	25.59	-112.63	-130.88
Alaska 2015	17 - 31 July, 2015	Gulf of Alaska	60.30	54.57	-132.86	-156.16
Pacific Mexico	3 - 23 August, 2010	Mexico's Pacific Coast	19.30	14.16	-101.15	-107.69
GOMECC-1	10 July - 4 August, 2007	U.S. East Coast<br>Gulf of Mexico	43.04	24.29	-68.40	-95.02
GOMECC-2	24 July - 13 August, 2012	U.S. East Coast<br>Gulf of Mexico	43.03	26.01	-68.52	-90.81
GOMECC-3	18 July - 21 August, 2017	Gulf of Mexico	30.03	18.83	-79.18	-97.73
ECOA	20 June - 23 July, 2015	U.S. East Coast<br>Canada Scotian Shelf	44.94	26.96	-61.73	-80.97
AZMP, Maritimes	20 - 24 September, 2015<br>7 - 15 May, 2016<br>5 - 28 September, 2017	Eastern Canadian Seas	64.20	45.78	-48.24	-65.88
AZMP, Newfoundland	8 - 28 July, 2017	Newfoundland and Labrador Regions	57.76	47.00	-43.00	-61.31
AZMP = Atlantic Zone Monitoring Program;						

Supplementary Table 2. Spatial cross-correlation coefficients between the ECOA data (2015) and GOMECC-1 data (2007) and between ECOA (2015) and GOMECC-2 (2012).

	SST	SSS	DIC	TA	TA/SSS	DIC/TA	pCO_2@25	pH@25	$\Omega_{arag@25}$
2015 vs. 2007	0.92	0.81	0.68	0.85	0.60	0.90	0.88	0.90	0.90
2015 vs. 2012	0.89	0.79	0.70	0.83	0.61	0.83	0.75	0.79	0.85

The original scattered data have been interpolated before running the cross-correlation analyses due to the differences in station locations. The p-value is less than 0.0001 for each coefficient. pCO_2, pH and Ω_{arag} are only analyzed based on the temperature normalized data.

Supplementary Table 3a. Statistical correlations (r values) between various parameters. N is number of observations. Note red color indicates a *p* value larger than 0.05 or not statistically significant.

	N	SST & TA	SST & DIC	SST & pCO_2	SST & pH	SST & Ω	SST & DIC/TA		
Atlantic Coast	548	0.602	0.046	0.356	-0.255	0.886	-0.886		
Gulf of Mexico	260	-0.037	-0.244	-0.107	0.098	0.475	-0.448		
Pacific Coast	610	-0.029	-0.292	-0.164	0.123	0.542	-0.500		
	N	SSS & TA	SSS & DIC	SSS & pCO_2	SSS & pH	SSS & Ω	SSS & DIC/TA		
Atlantic Coast	548	0.989	0.794	-0.123	0.290	0.839	-0.811		
Gulf of Mexico	260	0.608	0.666	0.726	-0.796	-0.416	0.195		
Pacific Coast	610	0.974	0.850	0.378	-0.332	0.005	0.148		
	N	DO & TA	DO & DIC	DO & pCO_2	DO & pH	DO & Ω	DO & SST	DO & SSS	DO & DIC/TA
Atlantic Coast	411	0.212	0.045	-0.300	0.336	0.264	0.181	0.261	-0.313
Gulf of Mexico	255	0.065	-0.139	-0.278	0.150	0.271	0.051	0.189	-0.436
Pacific Coast	524	-0.004	-0.440	-0.795	0.838	0.823	0.226	-0.046	-0.837
	N	DIC/TA & pCO_2		DIC/TA & pH		DIC/TA & Ω			
Atlantic Coast	548	0.063		-0.192		-0.996			
Gulf of Mexico	260	0.716		-0.670		-0.927			
Pacific Coast	610	0.860		-0.899		-0.985			

Supplementary Table 3b. ***p* values for all statistical correlations in Table 2a.** N is number of observations. Note red color indicates *p* value larger than 0.05 or not statistically significant.

	N	SST & TA	SST & DIC	SST & pCO_2	SST & pH	SST & Ω	SST & DIC/TA		
Atlantic Coast	548	0.0000	0.2866	0.0000	0.0000	0.0000	0.0000		
Gulf of Mexico	260	0.5544	0.0001	0.0857	0.1151	0.0000	0.0000		
Pacific Coast	610	0.4682	0.0000	0.0000	0.0024	0.0000	0.0000		
	N	SSS & TA	SSS & DIC	SSS & pCO_2	SSS & pH	SSS & Ω	SSS & DIC/TA		
Atlantic Coast	548	0.0000	0.0000	0.0039	0.0000	0.0000	0.0000		
Gulf of Mexico	260	0.0000	0.0000	0.0000	0.0000	0.0000	0.0016		
Pacific Coast	610	0.0000	0.0000	0.0000	0.0000	0.8986	0.0002		
	N	DO & TA	DO & DIC	DO & pCO_2	DO & pH	DO & Ω	DO & SST	DO & SSS	DO & DIC/TA
Atlantic Coast	411	0.0000	0.3677	0.0000	0.0000	0.0000	0.0002	0.0000	0.0000
Gulf of Mexico	255	0.2985	0.0266	0.0000	0.0167	0.0000	0.4143	0.0025	0.0000
Pacific Coast	524	0.9198	0.0000	0.0000	0.0000	0.0000	0.0000	0.2912	0.0000
	N	DIC/TA & pCO_2		DIC/TA & pH		DIC/TA & Ω			
Atlantic Coast	548	0.1427		0.0000		0.0000			
Gulf of Mexico	260	0.0000		0.0000		0.0000			
Pacific Coast	610	0.0000		0.0000		0.0000			
